# High conversion synthesis of <10 nm starch-stabilized silver nanoparticles using microwave technology

**DOI:** 10.1038/s41598-018-23480-6

**Published:** 2018-03-23

**Authors:** Shishir V. Kumar, Adarsh P. Bafana, Prasad Pawar, Ashiqur Rahman, Si Amar Dahoumane, Clayton S. Jeffryes

**Affiliations:** 1 Nanobiomaterials and Bioprocessing Laboratory (NABLAB), Dan F. Smith Department of Chemical Engineering, Lamar University, PO Box 10051, Beaumont, TX 77710 USA; 2 School of Biological Sciences & Engineering, Yachay Tech University, Hacienda San José s/n, San Miguel de Urcuquí, 100119 Ecuador; 3 Center for Advances in Water & Air Quality, Lamar University, 211 Redbird Ln. Box 10888, Beaumont, TX 77710-0088 USA

## Abstract

A microwave reaction to convert 99 ± 1% of Ag^+^ to silver nanoparticles (AgNPs) of size <10 nm within 4.5 min with a specific production rate and energy input of 5.75 mg AgNP L^−1^ min^−1^ and 5.45 W mL^−1^ reaction volume was developed. The glucose reduced and food grade starch stabilized particles remained colloidally stable with less than a 4% change in the surface plasmon resonance band at 425–430 nm at t > 300 days. TEM determined the size of AgNPs, while TEM-EDS and XRD verified elemental composition. The conversion was determined by inductively coupled plasma atomic emission spectroscopy (ICP-AES) and thermal gravimetric analysis (TGA). Additionally, the required silver to starch input mass ratio, 1.0:1.3, to produce colloidally stabilized AgNPs is significantly reduced compared to previous studies. The antibacterial activity of freshly prepared AgNPs and AgNPs aged >300 days was demonstrated against *E*. *coli* as determined by agar diffusion assays. This result, corroborated by spectrophotometric and TEM measurements, indicates long-term colloidal stability of the product. Thus, this study sustainably produced antibacterial AgNPs from minimal inputs. In the broader context, the current work has quantified a sustainable platform technology to produce sphere-like inorganic nanoparticles with antimicrobial properties.

## Introduction

Nanomaterials find applications in the areas of electronics, specialty chemicals and biotechnology, to name a few^1^. Metallic nanoparticles in the form of colloidal nanospheres have been of continuous research interest, since their intrinsic properties can be finely tuned by changing parameters such as diameter, chemical composition, bulk structure, surface chemistry and crystallinity^2,3^. Specifically, silver nanoparticles (AgNPs) have gained attention as they show potential applications in various fields, such as the environment, catalysis, optics, electronics and as antimicrobial agents^4–6^. However, strategies for benign synthesis of nanoparticles (NPs) should be considered to decrease the use of toxic reagents and solvents and reduce the generation of undesirable by-products^7^. Accordingly, “green chemistry”, which values the design of reaction pathways with benign reagents, aqueous solvents and few by-products, is preferred to conventional strategies^8^. To justify the method’s usability, a need for quantification and optimization of energy and substrate inputs is required, which has been reported for the first time in this work.

The use of sugars as reducing agents for the synthesis of inorganic NPs have proven effective for the synthesis of silver, copper and gold based nanoparticles, and metal oxide hollow spheres^9–13^. Additionally, sugars are cost effective, harmless to the environment^14^ and preclude the use of conventional reagents, such as sodium borohydride^1^ or N,N-dimethylformamide^15^, which further consolidates their position as one of the best choices for sustainable NP synthesis. It is, however, reported that the bactericidal properties of the AgNPs are size dependent, with AgNPs having diameters of ~1–10 nm being most effective^16^. Therefore, applications of NPs are dependent on their size, shape and stability^16,17^. To this end, NPs must be stabilized by a capping agent, for which starch is a suitable candidate as it is easily available^18^, benign^14^, and inexpensive^14^. However, life cycle analysis (LCA) of starch-based AgNPs indicate detrimental environmental impacts from the production of starch despite its biological origin and relatively benign characteristics when compared to chemical capping agents^19^. Therefore, the NP synthesis in this study was designed to achieve NP colloidal stability while using the minimal amount of starch. Furthermore, with the view to support environmental sustainability and reduce the steps of purification involved in production of laboratory grade soluble starch, we opted to make use of readily available food-grade starch.

In this study, we aimed to synthesize spherical AgNPs using glucose as the reducing agent and starch as the stabilizing agent. Most synthesis methods use a heat source to reduce the reaction time, improve kinetics and achieve higher yields and selectivity^10^. To increase AgNP syntheses rates, we used a laboratory-grade microwave, which allows better tuning of process parameters by virtue of easily adjusted heating rates and real-time process monitoring^20^. This control enables, as a platform technology, accurate and reproducible conditions to create a broad range of NPs. Microwave-assisted synthesis of AgNPs with starch and glucose have been studied^13^, but there was no mention of conversion rate or specific power for the AgNPs produced. Thus, to build upon the previous method, we have parameterized and standardized an AgNP production strategy based on energy and substrate inputs. Starch acts as both a stabilizing agent and a template to produce sphere-like shapes with small particle diameters^15^. Templates provide a constrained environment during nanoparticle growth and hence have a significant effect on the morphology of the as-produced NPs^21^. This is an important parameter since AgNPs of less than 10 nm have shown enhanced antimicrobial activity^16^, but previous reports using microwave-assisted and starch stabilized syntheses have only obtained AgNPs in the range of 10–34 nm^22^. Therefore, the objectives of this study are to create a microwave-assisted synthesis process using minimal and environmentally benign inputs that produce colloidally stabilized AgNPs of size less than 10 nm and possessing antibacterial properties against *E*. *coli*. This study presents new information on quantification of conversion, optimization of process inputs, for AgNP synthesis than previously reported by similar methods^23^.

## Results and Discussion

**Figure 1:**
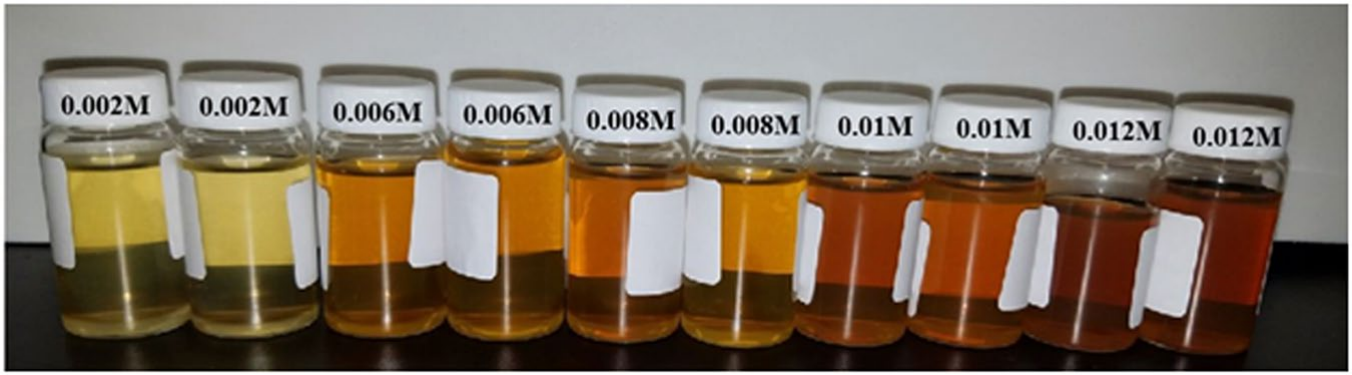
Digital images for the freshly prepared colloids of AgNPs, via a microwave-assisted route.

Digital images of the as-synthesized AgNPs are presented in Fig. 1. First, there is a color change from transparent before the exposure of the reaction media to the microwave irradiation to yellow-brown depending on the composition of the sample once the reaction occurred. This indicates clearly the reduction of cationic silver to its metallic counterpart and the formation of AgNPs. The reaction vials were not turbid nor did they contain any visible aggregates or precipitates, so the as-synthesized AgNPs were assumed to form stable colloids. The evolution of the color from pale yellow to dark brown is related to the initial concentration of AgNO_3_. By increasing its amount, the colloid turned brown, evidencing the formation of AgNPs at a higher concentration.

### Evaluation of production and stability of AgNPs by UV-Visible spectrophotometry

**Figure 2:**
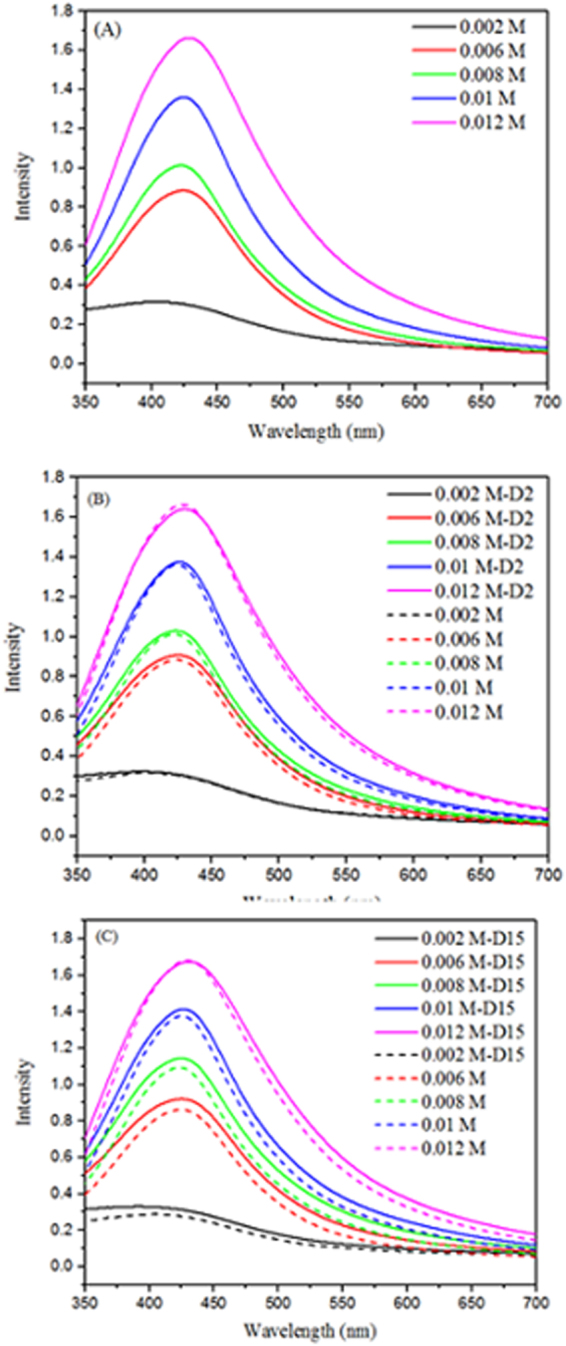
UV-Vis spectra of AgNP colloids, prepared by reacting AgNO_3_, glucose and starch under microwave irradiation: (**A**) Averaged spectra of duplicate samples; (**B**) Comparison with day-2 (D2); and (**C**) Comparison with day 15 (D15). The molar concentrations of AgNO_3_ were 0.002 M, 0.006 M, 0.008 M, 0.01 M and 0.012 M.

The Surface Plasmon Resonance (SPR) band, due to the collective oscillation of the electrons at the surface of metallic silver, is observed in all the samples in the range of ~420–430 nm^24^ (Fig. 2). This establishes again the synthesis of AgNPs starting from AgNO_3_ in the presence of glucose and starch and under microwave irradiation. With the increase in the concentration of AgNO_3_ and glucose, there is an increase in the intensity of the SPR band. This indicates more AgNPs are formed with increasing AgNO_3_ up to 0.012 mol L^−1^. Furthermore, the presence of a single SPR peak indicates the formation of sphere-like nanoparticles^25^. At concentrations higher than 0.012 mol L^−1^, the amount of starch may have been insufficient to stabilize the reduced Ag and hence AgNPs precipitated along with a fall in the absorbance, as depicted in the supplementary file (Figure S2). We observed no stable NPs above an Ag:starch mass ratio of 1.0:1.3, indicating the minimum starch required for particle stability.

The value of λ_max_ changes negligibly as the concentration of AgNO_3_ increases from 0.002 mol L^−1^ to 0.012 mol L^−1^, leading us to infer that the AgNPs present in the different samples of different AgNO_3_ concentrations possess similar shapes and sizes. We observe less than a 2% decrease in the absorbance intensity of the colloidal AgNPs after 15 days (Fig. 2(C)), which goes on to remain stable with a less than 4% decrease in absorbance even after t > 300 days, (Figure S9). We can hence state that the AgNPs remain colloidally stable over time without any precipitation or aggregation, thereby signifying the integrity of the product.

**Figure 3:**
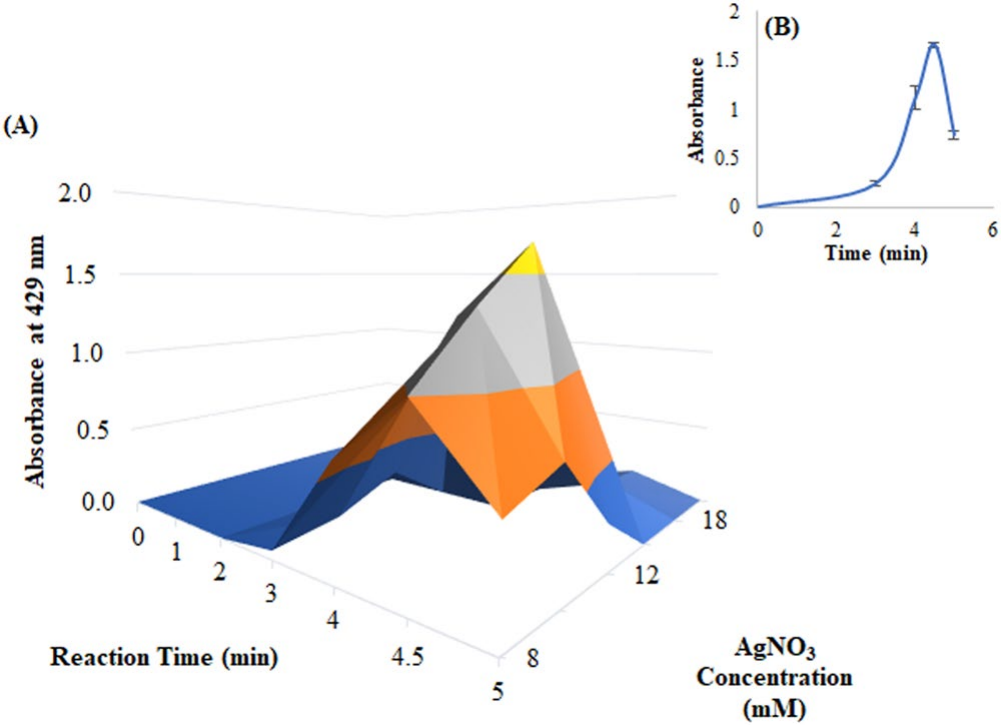
(**A**) Surface plot showing the growth curve for the absorbance of the SPR band at 429 nm as a function of reaction time and AgNO_3_ concentration; (**B**) SPR excitation at 429 nm for the 12 mM AgNO_3_ synthesis with respect to reaction time.

The production of colloidally stable AgNPs, measured by the absorbance of the SPR band at 429 nm as a function of reaction time and AgNO_3_ concentration, is presented in Fig. 3(A). The reaction temperature was linearly dependent upon reaction time and reaction vessel pressure was maintained above the saturation pressure. The total energy input was also linearly dependent on time and hence did not qualify as an independent variable. A power input below the maximum would have increased synthesis times and subsequently led to increased energy losses due to heat transfer from the reaction vessel. Therefore, maximum power was used as it was most efficient. Aqueous phase reactions in subcooled liquids poorly depend on pressure, so pressure effects were assumed to be negligible. The concentration of starch was fixed in the reactions, so the AgNO_3_ and AgNO_3_:starch ratio was linearly dependent, and the ratio of glucose to AgNO_3_ was fixed. Therefore, the primary independent variables examined in this work were reaction time and AgNO_3_ concentration.

Synthesis reaction times over a range of AgNO_3_ concentrations were tested and the maximum for production of stabilized particles was found to be at a reaction time of 4.5 min, independent of concentration (Fig. 3). The SPR excitation at the characteristic wavelength of 429 nm for the sample with the concentration of 0.012 mol L^−1^ cationic silver with respect to reaction time is shown in Fig. 3(B), which reaches a maximum value at 4.5 min. At longer reaction times particle instability was observed, presumably because of the degradation of the starch capping agent^26^. The production of stable AgNPs at the optimal 4.5 min reaction time as a function of AgNO_3_ concentration determined that a 0.012 mol L^−1^ input was optimal. Lower concentrations produced suboptimal quantities of AgNPs, while at higher concentrations the NPs lacked stability due to insufficient capping agent because of the decreasing AgNO_3_:starch ratio. Full experimental replicates of our duplicate reactions were carried out and the optimized 0.012 mol L^−1^ synthesis was found to have less than a 15% difference in SPR band absorbance (Figure S10).

### Morphology, particle size analysis and crystal structure identification

**Figure 4:**
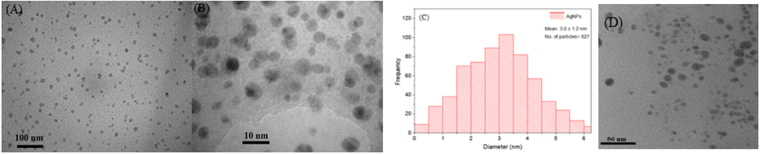
(**A**,**B**) TEM micrographs of the AgNPs obtained with 0.012 M AgNO_3_, under 2 different magnifications; (**C**) Mean particle size obtained from the TEM micrograph of (**A)**; (**D**) TEM on the replicate sample for AgNPs obtained with 0.012 M AgNO_3_.

Transmission Electron Microscopy (TEM) of the 0.012 mol L^−1^ AgNO_3_ sample showed well-dispersed, mostly spherical AgNPs (Fig. 4(A,B)) with a mean particle size of 3.0 ± 1.2 nm (n = 627) as depicted in Fig. 4(C), determined using ImageJ software. The AgNPs remained colloidally stable even after t  > 300 days as is evident from the TEM images shown in Figure S11.

TEM micrograph of the new batch of AgNPs is presented in Fig. 4(D). The mean particle size from this batch at a concentration of 0.012 mol L^−1^ AgNO_3_ was determined to be 5.4 ± 4 nm (n = 200), which confirms the method’s ability to consistently produce <10 nm AgNPs. Besides, the small size of the AgNPs may enable them to be used as seeds for seed mediated synthesis of different metal NPs as reported in previous studies^21,27^. TEM results prove the significance of this work and to our knowledge is the first reported instance for the production of starch-stabilized AgNPs of <10 nm size, through a microwave-assisted procedure. These AgNPs, hence, were expected to show antibacterial propoerties^16^, as shown below in our results.

**Figure 5:**
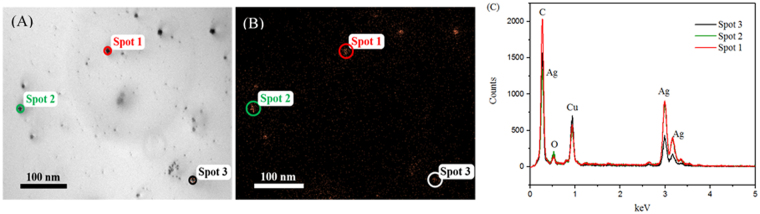
(**A**) Spot analysis showing field of view with spots; (**B**) Mapping of Ag for the 0.012 M of AgNO_3_ reacted to form AgNPs; and (**C**) EDS spectrum for spot analysis showing elemental silver.

To confirm the chemical composition of the particles seen using TEM, we carried out the elemental analysis using X-Ray Energy Dispersive Spectroscopy (TEM-EDS) on the three spots shown in Fig. 5(A), the silver on the spots shown in Fig. 5(B). The spectrum for each of these spots show the presence of silver, as can be seen in Fig. 5(C). In addition, the mapping on our sample highlights hotspots of silver whose composition was previously confirmed using EDS (Fig. 5). Moreover, the signal due to the presence of silver arises and is distributed where AgNPs are present in the corresponding STEM image (red signal in Fig. 5(C)). Taken together, the TEM image and EDS spectra, we conclude that our synthesis method yielded stable colloidal AgNPs.

**Figure 6:**
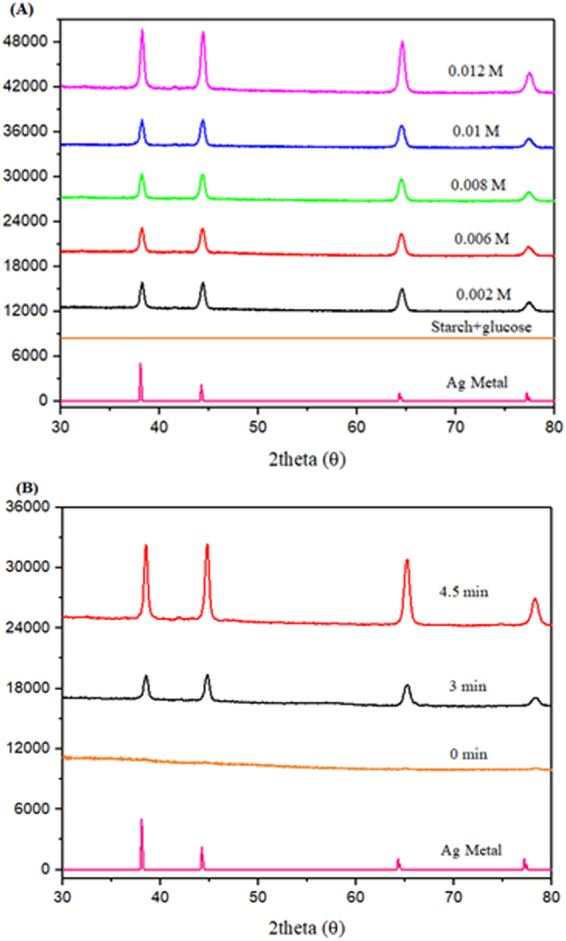
(**A**) XRD pattern showing characteristic peaks for metallic silver of AgNP samples obtained from initial concentrations of AgNO_3_: 0.002 M, 0.006 M, 0.008 M, 0.01 M and 0.012 M, compared to a reference (Ag Metal). The control is made of starch + glucose; (**B**) Growth of AgNPs with time from t = 0 to t = 4.5 min.

The X-Ray Diffraction (XRD) pattern presented in Fig. 6(A) shows the characteristic 2θ peaks at 38.07, 44.25, 64.36 and 77.30° for silver nanoparticles, corresponding to the (111), (200), (220) and (311) planes of the face-centered cubic (fcc) structure of metallic silver (JCPDS files no. 03–0921) as obtained by Manikprabhu and Lingappa^28^. Similar results were obtained by Gao *et al*.^29^, thus validating the presence of metallic silver. Fig. 6(B) shows the conversion of Ag^+^ to AgNPs as from t = 0 to t = 4.5 min. We observed only peaks for metallic silver which indicates that in our synthesis mechanism that cationic silver proceeds directly from cationic silver to its metallic counterpart without an intermediate crystalline phase, such as a silver salt or silver oxide. The formation of AgNPs increased with time as the shapes were templated by the starch capping agent^29^.

### Reaction conversion and optimization of inputs

**Figure 7:**
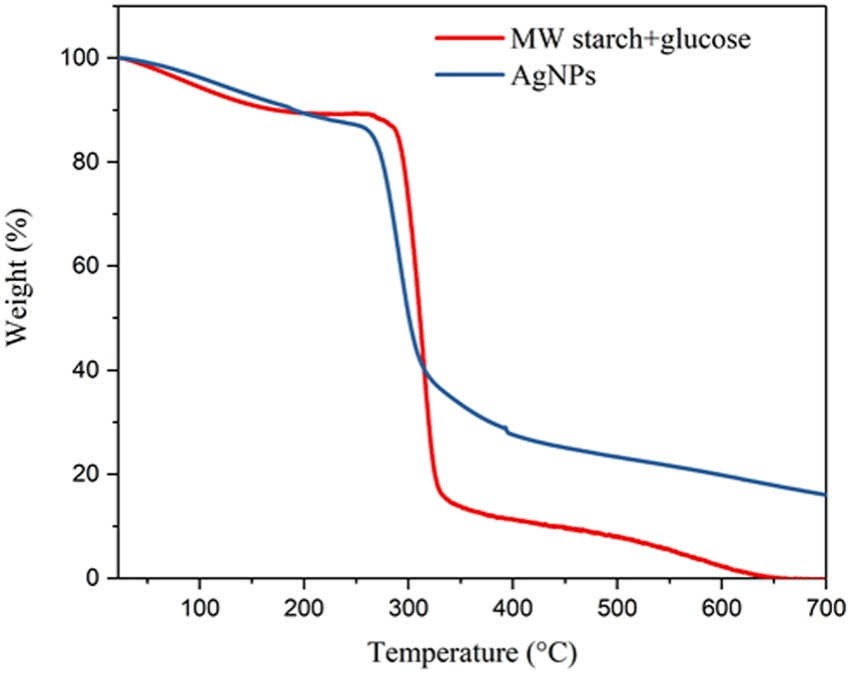
TGA curve for 0.012 M of AgNO_3_ reacted to form AgNPs and Ag-free control of starch and glucose.

Thermogravimetric analysis (TGA) of the dried AgNPs and that of the Ag-free control reaction composed of only starch and glucose are shown in Fig. 7. There is a sharp drop in weight at 314 °C for the starch and glucose mixture, which gradually goes to zero at 650 °C and is zero till the end of the run at 700 °C. A similar onset of sharp decrease was observed in the AgNP sample. However, at the end of the run at 700 °C, there was about 16 wt% of sample still remaining which represents the AgNP part of the sample. While the mass of the no-Ag, control sample went to zero by 650 °C, the AgNP sample continued to lose mass until 700 °C. This phenomenon has been observed in previous work and it was determined that surface interactions between bio-organic compounds, in our case starch, can be stabilized on the NP surface, delaying their volatilization^30^. There was negligible Ag^+^ cation in the TGA sample because unreacted AgNO_3_ is not precipitated in ethanol (solubility 0.75 kg Ag^+^ L^−1^ ethanol). Thus, we validate the presence of starch-stabilized AgNPs as the organics volatilize at higher temperatures, leaving behind only silver.

**Figure 8:**
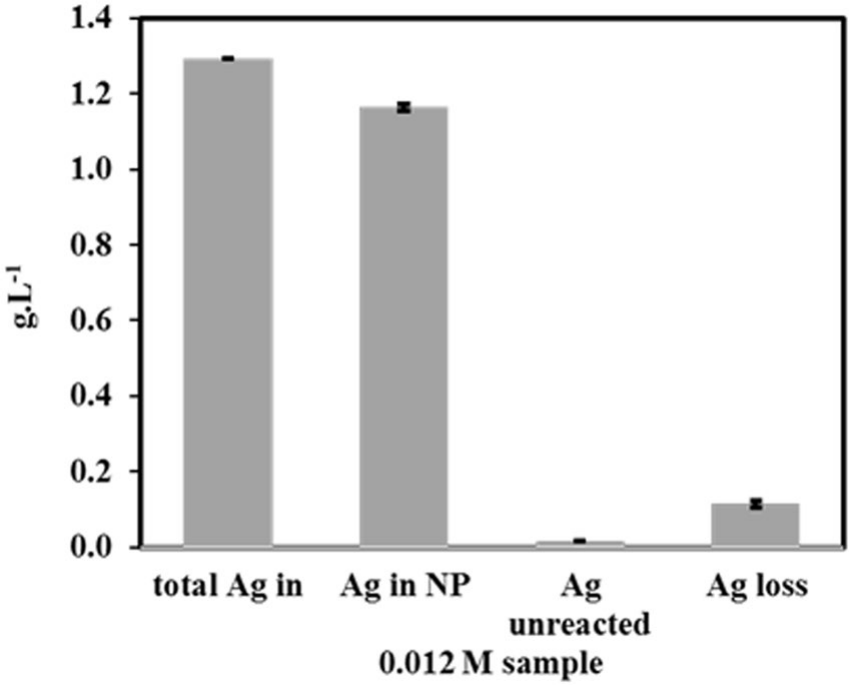
Conversion for the reaction with 0.012 M silver concentration, using ICP- AES results. “Total Ag in” represents the total charge of Ag L^−1^ of reaction volume, “Ag in NP” represents the Ag in the AgNPs, “Ag unreacted” represents the Ag^+^ found in the reaction supernatant and the “Ag loss” is the Ag unaccounted for in the ICP-AES measurements when compared to the total Ag charged to the reaction vessel, as described in the text.

The conversion of Ag^+^ to AgNPs was calculated to be 99 ± 1% based on the elemental analysis performed using the inductively coupled plasma atomic emission spectroscopy (ICP-AES). Fig. 8 shows the quantity of Ag input to the synthesis reaction, total Ag detected in the reaction mixture after the synthesis, Ag in the supernatant after the removal of AgNPs, and Ag in the precipitated NPs. The Ag loss represents the difference in the mass of Ag input and mass of Ag detected after the synthesis reaction. Potential points of loss were volume lost during solution transfer and pipetting or adsorption of Ag^+^ or AgNPs to the vessel walls, centrifuge tubes or pipettes. Conversion was based on the mass balance between total Ag detected in the mixture after the synthesis, Ag^+^ in the supernatant and Ag in the precipitated NPs. Final TGA measurement of 16 wt% AgNP supports the ICP-AES measurements used to determine the 99 ± 1% conversion. This implies that our microwave-assisted method for the synthesis of AgNPs goes to completion, effectively consuming all silver substrate. Therefore, we followed some of the important principles of green chemistry as there is a high amount of desired product formed and side reactions are limited.

According to calculations found in the supplementary file (section S2) the microwave-assisted reaction system used 29.43 kJ of energy for a single 20 mL synthesis reaction. A direct comparison of energy usage between a microwave-assisted reaction system and a synthesis method using conductive heating is difficult because of the many types of conductive heating systems employed. Experiments in the author’s lab (unpublished data) required 1 h on a hotplate (1000 W) to synthesize similar AgNPs, compared to 4.5 min under microwave conditions. However, one previous study reports a comparison between conductive heating and the microwave-assisted formation of ZnO NPs, and determined the microwave route was four times faster^31^. Thus, current evidence points to a higher conversion per energy input using a microwave-assisted synthesis reaction. Indeed, there is already a vast body of work which describes the various production methods for AgNPs and starch-stabilized AgNPs synthesized via a microwave-assisted route^23^. However, we have innovated upon the work done in literature^13^ and optimized the parameters to produce sub 10 nm AgNPs fit for antibacterial applications^16^, with minimal material and energy utilization for glucose-produced and starch-stabilized AgNPs under microwave heating. Furthermore, the Ag:starch input mass ratio of 1.0:1.3 was between six to sixty times less than that reported in previous studies^19,29^, which could significantly reduce the environmental impact from the life cycle assessment (LCA) point of view, as well as reducing production costs^19^. Thus, the synthesis reaction in the current study uses less process inputs and therefore poses fewer environmental impact.

### Antibacterial activity of silver nanoparticles

**Figure 9:**
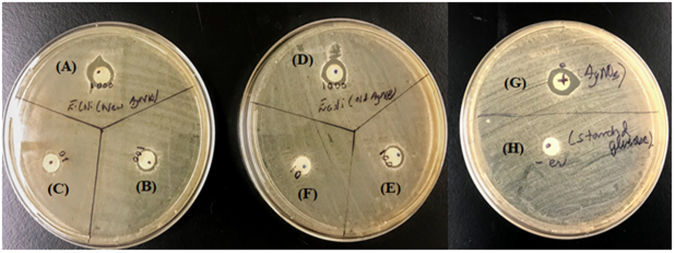
Antibacterial effect of freshly prepared AgNP doped discs at concentrations of (**A**) 1000, (**B**) 100 and (**C**) 10 µg mL^−1^ with kill zones; antibacterial effect of AgNP doped discs with AgNPs at t > 300 days for concentrations of (**D**) 1000, (E) 100 and (**F**) 10 µg mL^−1^; (**G**) positive control using AgNO_3_ with silver concentration of 1000 µg mL^−1^ doped discs on an agar diffusion assay plate; (**H**) negative control with microwaved reaction product of starch and glucose without any silver doped discs on an agar diffusion assay plate.

Fig. 9(A–C) and (D–F) show the antibacterial effect of freshly prepared and t > 300 days old AgNPs, respectively. The kill zone (diameters provided in Table 1), which is the space around the AgNP doped discs showing no bacterial growth after 24 h incubation at 37 °C, is apparent in both the freshly prepared AgNPs as well as the t > 300 days old sample, which tells us that the antibacterial activity of the AgNPs remain present even after t > 300 days. The ICP-AES and TGA results (Figs 8, 7, respectively) that show 99 ± 1% conversion confirm that the microwave synthesis reaction goes to near completion under the conditions used to produce the AgNPs tested for anti-bacterial activity, indicating the antibacterial activity is due to the NPs and not residual Ag^+^. This is supported by the fact that the antibacterial activity may be attributed to the association of AgNPs with the bacterial cell membrane causing physical damage to the cells possibly through pitting^32,33^.

**Table 1:** Kill zone diameters for AgNPs of varying concentrations and ages.

Ag Concentration (µg mL^−1^)	Sample Age (days)	Kill Zone Diameter (mm)
1000	1	11.5
1000	320	10.3
100	1	8.7
100	320	8.4
10	1	7.5
10	320	7.1

The AgNPs at t > 300 days showed kill zones of comparable size (kill diameters of 93 ± 3%) to those of new AgNPs. This signifies that both the AgNPs (new ones as well as AgNPs at t > 300 days) have similar antibacterial activity. The UV-Vis spectra (Figure S9) and TEM images (Figure S11) are provided for the AgNPs at t > 300 days, which indicate that the antibacterial activity is likely due to the presence of AgNPs. However, to further understand the antibacterial activity, we carried out the ICP-AES on the AgNPs which were aged over t > 300 days. We observed an increase in the amount of silver in the supernatant by 25 mass% which likely corresponds to cationic silver from surface dissolution of the AgNPs. This hints towards the possible antibacterial effect due to cationic silver through the oxidative dissolution of Ag^0^ to Ag^+^ ^34,35^. In the light of the information presented here, we may infer that the antibacterial activity of AgNPs at t > 300 days is possibly due to a combined physical interaction of the NPs with the cell membrane as well as the oxidative dissolution of Ag^0^ to Ag^+^. Thus, in totality the antibacterial activity may correspond to the “nanoparticle effect” as stated in literature^32^. This implies that the differently aged AgNPs have similar antibacterial activities since the mechanism of action may be the same for AgNPs as well as cationic silver, although a comparison of the kill zones (Fig. 9) that the antibacterial activity even after t > 300 days is still predominantly due to the physical interaction of NPs with the cell surface.

The positive control (Fig. 9(G)) using AgNO_3_ with a silver concentration of 1000 µg mL^−1^ showed a kill zone of 12.5 mm, establishing the ability of AgNPs of an equivalent concentration to have nearly the same antibacterial activity as that of cationic silver, while the negative control (Fig. (9)) a microwaved reaction product of starch and glucose without any silver, showed no apparent kill zone, thereby validating the presence of silver being the antibacterial agent. The sterility control with AgNPs evenly distributed over the agar plate showed no growth, thus confirming that the colloidal AgNPs used to test the antibacterial properties were uncontaminated at the time of use.

## Methods

### Synthesis of silver nanoparticles

For the preparation of colloidal silver nanoparticles, AgNO_3_ (20% w/v) from Aqua Solutions (Deer Park, Texas, USA) was the precursor, D(+) glucose from Alfa Aesar was the reducing agent, food grade corn starch (Hill Country Fare, H-E-B, San Antonio, Texas, USA) was the NP stabilizing agent, and deionized water (DIW, 18.2 MΩ) was the solvent.

The preparation of AgNPs and the associated control experiments are described in Table 2. For AgNP synthesis, 10 mL of 3.4 g L^−1^ starch solution and 6 mL of glucose solution were added into 4 mL of AgNO_3_ solution in 40 mL XP 1500 TFM^®^ Fluoropolymer lined vessels. The starch concentration was kept constant for all experiments while the molar ratio of AgNO_3_:glucose was kept constant at 1.0:1.5 to increase conversion of Ag^+^ ions to Ag^0^ and promote the subsequent formation of AgNPs. The optimized reaction time was 4.5 min at a power of 1200 W in a CEM MARS 5 microwave reaction system equipped with reaction vessel temperature and pressure sensors. The reactions reached an average temperature and pressure of 128 ± 5 °C and 287 ± 21 kPa absolute. The volume of all simultaneous reactions in the microwave system was 220 mL (10 reaction vessels plus the instrument control vessel), bringing the specific power input to 5.45 W mL^−1^, for a total of 1473 J mL^−1^. All reactions were done in duplicate.

**Table 2:** Reaction parameters for the production of AgNPs, total reaction volume per 20 mL synthesis reaction.

Reactant name and concentration	Starch (g L^−1^)	Glucose (M)	AgNO_3_ (M)
Microwave synthesis	1.7	0.003 0.009 0.012 0.015 0.018	0.002 0.006 0.008 0.01 0.012
Glucose control	1.7	0	0.012
Starch control	0	0.018	0.012
Only AgNO_3_	0	0	0.012

The time parameter was optimized for maximum production of AgNPs at the given concentrations and are presented in Table 2. To reach the parameters presented in Table 2, we carried out experiments to evaluate the effects of time, concentrations of AgNO_3_ and starch, details of which are provided in the supplementary material (Table S1, Figures S1–S6). The controls are described below.

To study the effect of glucose on the reduction of cationic silver, we carried out a reaction with 10 mL of 3.4 g L^−1^ starch solution along with 4 mL of 0.06 mol L^−1^ AgNO_3_ solution, but the glucose solution was replaced by the same volume of DIW (Figure S4). Another control was performed with 4 mL 0.06 mol L^−1^ AgNO_3_ in DIW without glucose or starch. The final control was carried out to study the role of starch in the reduction process by taking 4 mL of AgNO_3_ and 6 mL of glucose solution in the vessel at a concentration of 0.01 mol L^−1^ and 0.06 mol L^−1^, respectively, without any starch (Figure S4). The produced colloidal AgNPs were stored in dark at 4 °C.

### Antibacterial activity of silver nanoparticles

The antibacterial effect of the as-synthesized AgNPs was tested on *E*. *coli* ATCC# 26, a gram- negative species. The composition of the culture media was 8 g L^−1^ of Difco™ Nutrient Broth (containing 3.0 g of beef extract with 5.0 g of peptone) in tap water at a final pH of 6.8. Agar diffusion assays were done using the same media with the addition of 15 g L^−1^ dehydrated agar powder obtained from Fisher Scientific.

Glycerol frozen *E*. *coli* were initially introduced into 2.5 mL of sterile culture media in 16 × 150 mm test tubes under aseptic conditions. This broth culture was incubated in a shaking incubator for 24 h at 100 rpm and 37 °C. The optical density (O.D.) of the culture was measured using a Shimadzu UV- 1700 PharmaSpec UV-Vis spectrophotometer and fixed at 0.1 by serial dilutions with the culture media before plating.

The agar diffusion assay plates^36^ were prepared when the O.D. = 0.1 cultures were evenly applied to the agar plates of the culture media using sterile cotton swabs, to form a lawn of the bacterial culture. The culture plates were then incubated for 10 mins at 37 °C.

The AgNPs prepared a day before and those prepared 320 days earlier were both tested for their antibacterial activity. AgNP doped filter paper discs of 7 mm diameter were made by putting 20 µL of 10, 100 or 1000 µg mL^−1^ AgNP solution onto the filter paper discs, which were left to soak for 10 minutes before being applied to the agar diffusion assay plates. The positive control was 20 µL of AgNO_3_ solution with an Ag^+^ concentration of 1000 µg mL^−1^. The negative control was the microwave reaction product of starch and glucose without any silver. Plate sterility was verified by incubation at 37 °C for 24 h before the addition of any culture and lastly, a control to assess the interaction of AgNPs with the agar plates as well as its sterility was carried out by plating 1000 µg mL^−1^ colloidal AgNPs on an agar plate using a sterile cotton swab and incubating it at 37 °C for 24 h.

### Characterization techniques

#### UV-Visible spectrophotometry (UV-Vis)

Analyses of the as-produced colloidal AgNPs were carried out on microwaved and then cooled reaction mixtures using a Cary Varian 100 Bio UV-Vis Spectrophotometer. All the samples were analyzed in a 1.00 cm path length cuvette from 350 nm to 700 nm.

### Morphological analysis

The morphological studies were carried out using XRD and TEM. For XRD, 5 mL of colloidal AgNPs were mixed with an equal volume of ethanol (70%, VWR Analytics), to precipitate the AgNPs, and then vortexed for 2 min. The vortexed sample was then centrifuged at 1200 × *g* for 10 min. After centrifugation, the transparent supernatant was discarded and the pellet, which contained the AgNPs, was dried in the oven at 75 °C for 3 h. The dried pellet was crushed into a fine powder and analyzed using a Thermo Scientific ARL Equinox 100X-ray diffractometer operating with a Cu K $\alpha$ radiation source ($\lambda$ = 1.54 Å). The X-rays were generated at 41 kV and 0.9 mA power on a full 2θ range of 0°–116° for 20 min. As a control, the reaction mixture containing starch and glucose, but without any silver nitrate solution, was analyzed after it was microwaved at the same process parameters as for the synthesis of the AgNPs.

TEM samples were prepared by casting 20 µL of colloidal AgNP solution onto the surface of a PELCO^®^ TEM Grid Carbon Type- B (Ted Pella Inc., 3.05 mm O.D., 400 mesh, 0.4 × 2 mm single slot Cu) and allowed to dry in air for 24 h. TEM analyses were performed using a JOEL JEM- 1400 Plus Transmission Electron Microscope (120 kV, step- 1 kV, beam current- 69 µA) equipped with embedded Scanning Transmission Electron Microscopy (STEM) and an X-ray Energy Dispersive Analysis (EDS) probe provided by AMETEK EDAX. The STEM-EDS performed in STEM mode (120 kV, tilt angle 0°, spot size 3) included spot analyses for the sample as well as mapping to establish the presence of AgNPs in the sample, using EDAX TEAM™ software system.

### Thermogravimetric analysis

The silver composition of the ethanol precipitated colloidal AgNP solution was determined by TGA using a TA Instruments TGA-Q500 instrument. The heating rate was 20 °C min^−1^ and the experiments were performed in a nitrogen atmosphere at a flow rate of 60 mL min^−1^. The temperature range was from 22 °C to 700 °C. Samples were prepared per the XRD procedure.

### Evaluation of the synthesis conversion

ICP-AES (Shimadzu ICPE-9820, CCD detector) was used to quantify the conversion of Ag^+^ to AgNPs. The ICP was carried out with DIW as the blank and the detection of Ag was at a wavelength of 328.068 nm. ICP-AES measurements were carried out on the bulk mixture obtained after the microwave reaction, but before separation, to assess the total amount of silver (AgNPs plus unreacted silver cations) in the sample. Additionally, 5 mL of the AgNP colloid were centrifuged along with the same volume of 70% (v/v) ethanol (VWR Analytical) at 1200 × *g* for 10 min. The ICP-AES analysis for this supernatant determined the amount of unreacted silver cations present in the final mixture, thereby giving us the values to calculate the conversion of the reaction. Limits of detection for Ag were 0.3 µg L^−1^. Spectra were recorded in triplicate for duplicate synthesis reactions.

## Electronic supplementary material

Supplementary Information
