# Supplementary material for: High conversion synthesis of <10 nm starch-stabilized silver nanoparticles using microwave technology
Source: Sci Rep. 2018 Mar 23;8:5106. doi: 10.1038/s41598-018-23480-6 (PMC5865190; doi:10.1038/s41598-018-23480-6)
Supplement: Supplementary file 1 — Supplementary Information [file 41598_2018_23480_MOESM1_ESM.docx]

**Supporting Information**

**High conversion synthesis of < 10 nm starch-stabilized silver nanoparticles using microwave technology**

**Shishir V. Kumar^1^, Adarsh Bafana^1^, Prasad Pawar^1^, Ashiqur Rahman^1^, Si Amar Dahoumane^2^, Clayton S. Jeffryes^1*^**

*^1^Nanobiomaterials and Bioprocessing Laboratory (NABLAB), Dan F. Smith Department of Chemical Engineering, Lamar University, PO Box 10051, Beaumont, TX 77710, USA. cjeffryes@lamar.edu*

*^2^School of Biological Sciences & Engineering, Yachay Tech University, Hacienda San José s/n, San Miguel de Uruquí 100119, Ecuador. sdahoumane@yachaytech.edu.ec*

*Corresponding author

**S.1 Synthesis conditions screened for NP formation and stability**

The experiments listed in Table S1 were performed to assess the effect of various parameters on the synthesis of AgNPs. We carried out AgNP synthesis reactions with the same AgNO_3_:glucose ratio as described in the primary text while we changed the parameters for concentration of AgNO_3_, starch and time. In addition to the concentrations tested in the primary text, final concentrations as low as 0.00002 mol L^-1^ and as high as 0.06 mol L^-1^ were also tested, which yielded no apparent formation of AgNPs per the digital images (Figure S1) and formation of unstable NPs as described by the images and spectra (Figure S2). Similarly, we carried out experiments to check the effect of time and starch concentrations, wherein we looked at reaction times of 4 min and 5 min, and starch concentrations as low as 1.7 g L^-1^ and as high as 3.4 g L^-1^, respectively. With a decrease in reaction time we see that the reaction products appear to be paler (Figure S3), as compared to the products formed in the reactions described in the primary text, thus lesser amount of stable AgNPs are formed as the time was insufficient to carry out stabilization of the AgNPs while when the reaction time is increased the starch layer possibly breaks down and we get unstable AgNPs are obtained (Figure S4). Decreasing the concentration of starch led to no apparent AgNP formation as seen in Figure S5 and increasing the concentration led to formation of unstable AgNPs.

Based on the screening experiments, it was found that the response variable (SPR excitation intensity for AgNPs) was chiefly dependent on the reaction times and the concentration of the silver precursor, as other variables, such as temperature and pressure were a function of the energy input to the system, which in turn was set to the highest value lest we dissipate time and energy. Thus, we carried out a full factorial design for a two parameter system, with times at 3 min and 4.5 min and concentrations at 0.008 mol L^-1^ and 0.012 mol L^-1^. The SPR excitation response at 429 nm for these conditions were measured and a pareto plot was obtained which showed time to be the most important factor, followed by concentration (of silver input) as shown in Figure S8. At times higher than 4.5 min, the formed particles were no longer colloidally stable, hence any time greater than 4.5 min was not considered for the statistical estimation of the experiment’s parameters.

**S.2 Calculations**

*1. Calculation for energy consumed in the Microwave*

Total power of Microwave = 1200 W, No. of reaction vessels = 11, Volume of reaction mixture per reactor vessel = 20 mL

Energy per mL of reaction = 1200 W/(11*20mL) = 5.45 W/mL = 5.45 J/(s-mL)

Total energy required= (5.45 J/s-mL)*(20 mL)*(4.5 min)*(60s/min) = 29430 J =29.43 kJ

*2. Calculation for conversion of the 0.012 mol Ag L^-1^ reaction*

Total Ag in (g L^-1^) = 0.012 (mol L^-1^)$\times$107.8 (g mol^-1^) = 1.29 ± 0.00 (g L^-1^)

From ICP Calculation, Ag in NP (g L^-1^) = 1.16 ± 0.01 (g L^-1^)

Ag unreacted (g L^-1^) = 0.01 ± 0.00 (g L^-1^)

$$\% Conversion=\frac{\left( Total Ag in-Ag unreacted \right)}{Ag in NP}\times100=\frac{\left( 1.29 \pm0.01-0.01 \pm0.00 \right)}{\left( 1.29 \pm0.01 \right)}$$

$$\% Conversion=99 \pm1.0\%$$

**Spectrophotometric data**

Figure S9 shows a comparison between the absorbance intensity measurements on day 1 with that measured over 300 days after the sample was prepared. Figure S10 shows the UV-visible spectra for the replicate synthesis reactions introduced in the main document.

**TEM micrographs**

TEM micrographs of the AgNPs 320 days after synthesis are found in Figure S11. This sample was used in the antibacterial activity assays.

**S.3 Tables and Figures**

Table S1. Parameters evaluated to fix the values of AgNO_3_, time and starch for the experiment

| Parameter | Range |
| --- | --- |
| Concentration AgNO_3_ (mol L^-1^) | 0.00002 - 0.06 |
| Time (minutes) | 4 - 5 |
| Starch (g L^-1^) | 1.7 - 6.8 |


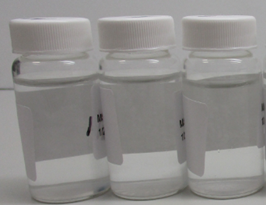


Figure S1: Results for concentrations of 0.00002 M, 0.00006, 0.001 M. There is no apparent formation of NPs.


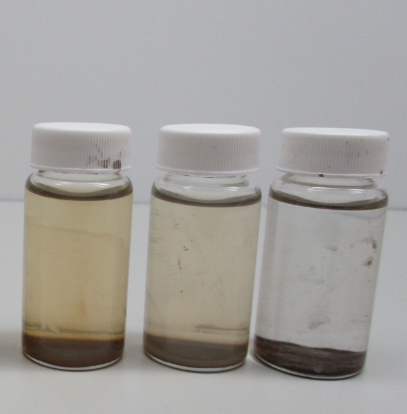

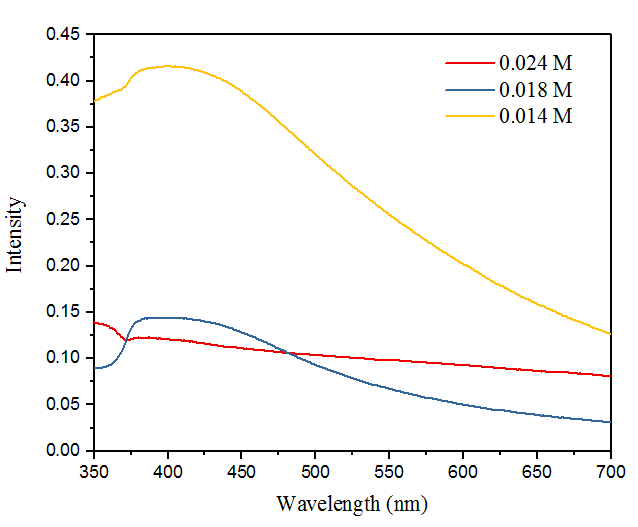
Figure S2: Results for AgNO_3_ concentration of 0.014 M, 0.018 M, 0.024 M. The synthesized particles were unstable (at right).


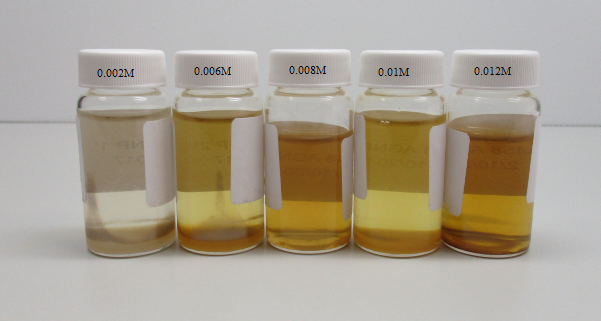
Figure S3: Digital images for reaction carried out at 4 minutes. Precipitate formation is visible.


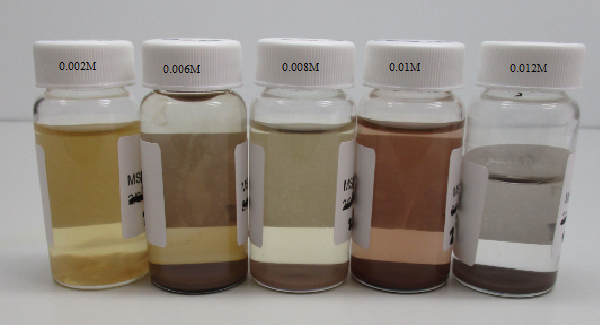
Figure S4: Digital images for reaction carried out at 5 minutes. Precipitate formation is visible.


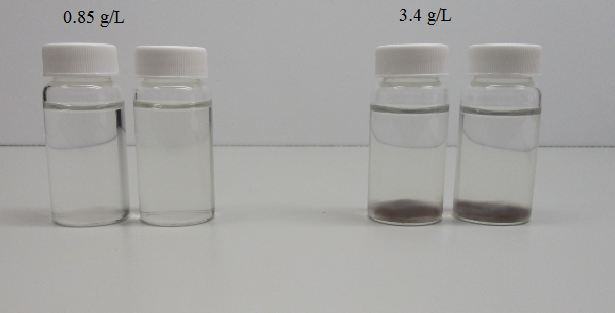
Figure S5: Digital images for reaction carried out with starch concentrations of 0.85 g L^-1^ and 3.4 g L^-1^. No visible reaction (left) and precipitate formation (right).


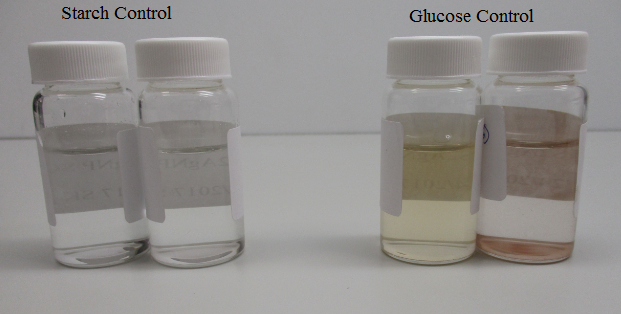


Figure S6: No formation is observed in the absence of starch (starch control, left) and very little reduction is observed in the absence of glucose (glucose control, right).


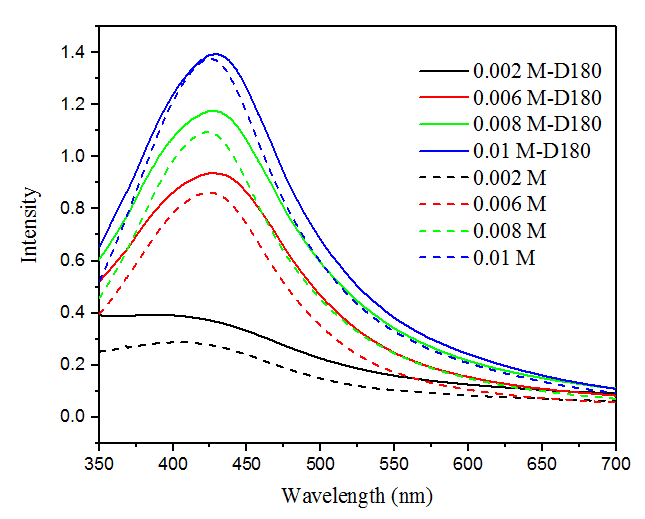
Figure S7: UV-vis spectra of the AgNPs after 180 days.


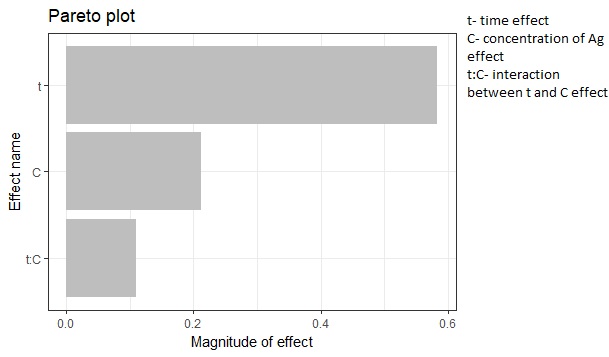
Figure S8: Pareto plot for the reaction parameters of time and concentration based on SPR excitation intensity as the response variable.


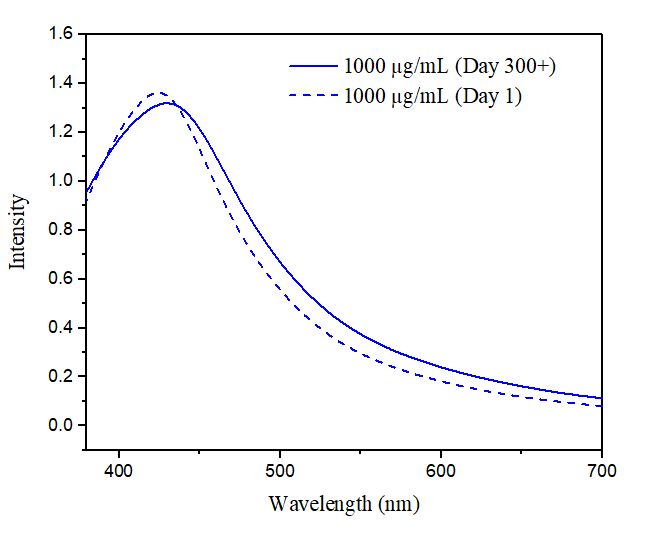
Figure S9: UV-Vis spectra for the samples used to show antibacterial activity of AgNPs at t=1 day and t >300 days.


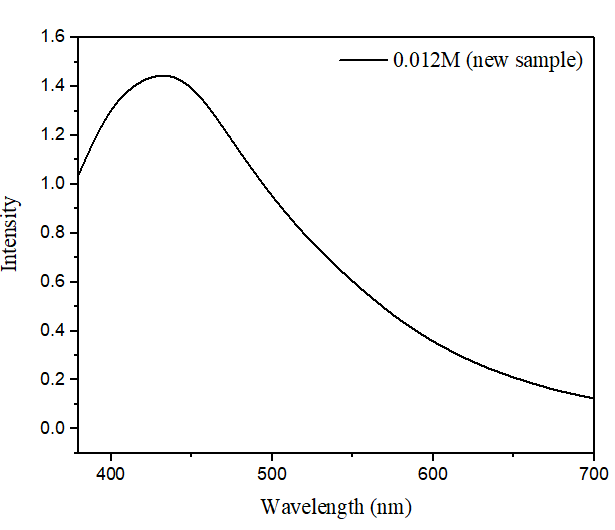
Figure S10: UV-Vis spectra for freshly prepared sample of 0.012M AgNPs


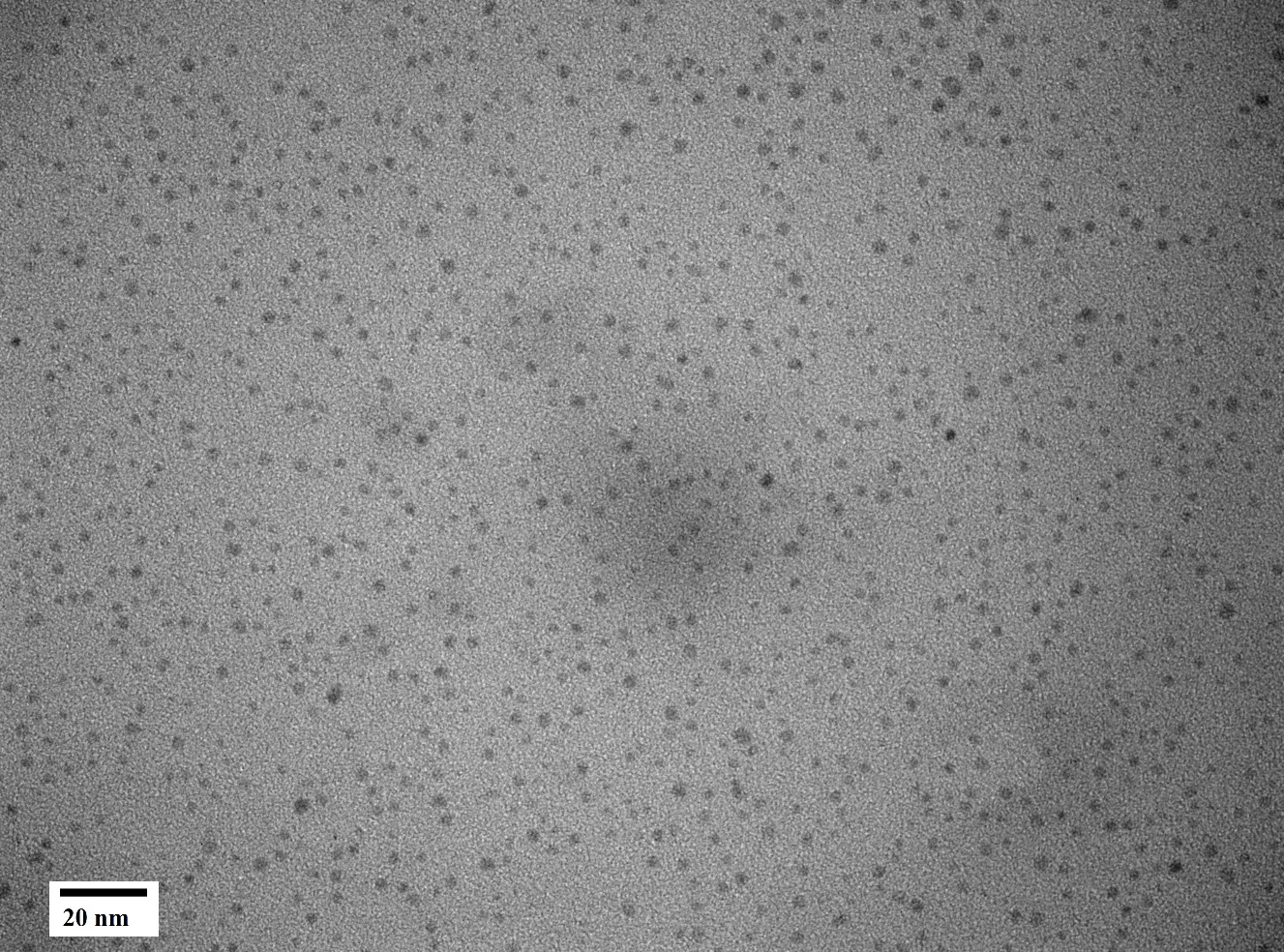


Figure S11: TEM micrograph of AgNPs at t >300 days. Particle size: 3.08 ± 1.30.
